# Seroprevalence of hepatitis C virus infection among individuals attending a private clinic in Luanda, Angola

**DOI:** 10.1186/s12879-026-13928-w

**Published:** 2026-07-01

**Authors:** José Fernando, Lourenço Quixari, Manuel Sangombe Epalanga, Mário Jorge Pascoal, Edson K. Cassinela, Sandra Inaculo, Edmundo Púcuta, Euclides Sacomboio, Domingos Jandondo, Joana M. K. Sebastião, Maria L. S. Silva, Ngiambudulu Francisco, Marta Pingarilho, Ana B. Abecasis, Joana Morais, Victor Pimentel, Cruz S. Sebastião

**Affiliations:** 1 Departamento de Investigação Científica, MEDIAG Análises Clínicas, Luanda, Angola; 2 Centro Nacional de Investigação Científica (CNIC), Luanda, Angola; 3 Instituto Superior de Ciências Policiais e Criminais (ISCPC), Luanda, Angola; 4 Institute of Health Sciences (ICISA), Agostinho Neto University (UAN), Luanda, Angola; 5 Centro de Investigação em Saúde de Angola (CISA)|Instituto Nacional de Investigação em Saúde (INIS), Luanda, Angola; 6 Faculdade de Medicina (FM), Universidade Katyavala Bwila (UKB), Benguela, Angola; 7 Global Health and Tropical Medicine, GHTM, LA-REAL, Instituto de Higiene e Medicina Tropical, IHMT, Universidade NOVA de Lisboa, Lisboa, Portugal

**Keywords:** Hepatitis C virus, Prevalence, Demographic characteristics, Angola

## Abstract

**Background:**

Hepatitis C virus (HCV) infection remains a major public health concern in sub-Saharan Africa (SSA), where epidemiological data are limited, particularly in countries such as Angola. Herein, we estimated the seroprevalence of HCV infection and demographic characteristics related to the infection in a large urban population in Luanda, the capital city of Angola.

**Methods:**

This was a retrospective cross-sectional analysis of existing laboratory records from 5,399 individuals using an immunoassay test for detecting anti-HCV antibodies, between 2020 and 2024 at the MEDIAG Laboratory, a private healthcare facility in Luanda, Angola.

**Results:**

Overall, 1.1% (57/5399) of participants were anti-HCV reactive. The mean age of reactive individuals was significantly higher than that of non-reactive participants (47.5 ± 15.7 vs. 37.3 ± 12.5 years, *p* < 0.001). Young individuals aged 20–30 years (AOR: 0.30, *p* = 0.003) or 31–40 years (AOR: 0.31, *p* < 0.001) showed lower odds of anti-HCV reactivity. Males showed numerically higher anti-HCV reactivity (AOR: 1.26, *p* = 0.407), but this association did not reach statistical significance. Between 2020 and 2024, overall anti-HCV reactivity among tested individuals changed slightly, from 0.9% to 1.1%, without statistical evidence of a temporal increase (*p* = 0.984). Among the 57 anti-HCV-reactive individuals, changes in the distribution of cases across age groups were observed over time, with the proportion in those under 20 years ranging from 0% to 5.9%, in those aged 20–30 years from 12.5% to 23.5%, and in those over 40 years from 50% to 64.7%, while a decrease was observed in the 31–40 years group (37.5% to 5.9%). Similarly, variations were observed by gender, with females decreasing from 75% to 47.1% and males increasing from 25% to 52.9%.

**Conclusion:**

Anti-HCV reactivity was more frequent among individuals aged over 40 years. The observed age and gender distributions may help identify groups for further surveillance and targeted screening strategies. However, these findings are based on individuals tested at a private healthcare facility and on anti-HCV antibody reactivity without HCV RNA confirmation, limiting inference regarding active infection and population prevalence. Further population-based studies with HCV RNA confirmation are needed to better estimate the true burden of HCV infection in Angola.

**Clinical trial number:**

Not applicable.

## Background

Hepatitis C virus (HCV) infection remains a significant public health concern globally with about 58 million people chronically infected and resulting in about 400,000 deaths each year from cirrhosis or hepatocellular carcinoma caused by HCV infection, according to the World Health Organization (WHO) [1, 2]. Sub-Saharan Africa (SSA) is one of the most affected regions, where limited surveillance, unsafe medical practices, and sociocultural factors contribute to the ongoing transmission of HCV [3].

HCV is often asymptomatic in its early stages, making active surveillance and early detection essential to prevent progression to cirrhosis or hepatocellular carcinoma [4]. Despite its growing urban population and extensive global data on HCV, Angola remains understudied in the context of HCV epidemiology. The capital city offers a relevant urban setting to explore the demographic distribution associated with HCV infection [5–9]. However, the lack of national screening programs and limited public awareness has resulted in insufficient data to guide public health interventions.

Currently, there are few studies on HCV and its respective epidemiological determinants conducted and published in different regions of Angola [5–9]. Therefore, the present study aimed to estimate anti-HCV seroprevalence and describe demographic characteristics associated with anti-HCV reactivity among individuals tested at a private clinical laboratory in Luanda, the capital city of Angola. This study contributes descriptive evidence on anti-HCV reactivity among individuals tested in Luanda, a country in Sub-Saharan Africa, thereby contributing to the limited epidemiological data available on HCV in the country [10].

## Materials and methods

### Study design and setting

This was a retrospective cross-sectional analysis of existing laboratory records from 5,399 individuals who received healthcare between January 2020 and December 2024 at the MEDIAG Clinical Analysis Laboratory, located in Luanda, the capital city of Angola. The study was explicitly framed as a clinic-based analysis of a high-risk, predominantly symptomatic population, rather than a general population or epidemiologically representative sample. We included individuals of any age who attended the MEDIAG Clinical Analysis Laboratory between January 2020 and December 2024 and who had anti-HCV antibody testing requested by a physician based on clinical suspicion. Records were included only when both serological results and key sociodemographic variables (age, gender, and year of consultation) were available. Information regarding the total number of records initially screened and the number excluded because of incomplete data was unavailable. The research was conducted by the National Centre for Scientific Research (CNIC), an institution engaged in multidisciplinary and interdisciplinary activities related to scientific research, experimental development, technology transfer, innovation, and technology-based entrepreneurship across various fields of knowledge. The study adhered to the ethical principles outlined in the Declaration of Helsinki. The study was approved by the General Management of the MEDIAG Clinic (approval number DTG-MED-05-007/2025). The requirement for informed consent was waived by the Clinical and Pedagogical Directorate of MEDIAG due to the retrospective nature of the study. All patient data was anonymised to ensure confidentiality and protect patient identity.

### Data/sample collection and laboratory procedure

Sociodemographic information, including age, gender, and year of consultation, was obtained from the medical records. HCV status was determined for all participants through the detection of anti-HCV antibodies (CLIA-HCV, Mindray), using a chemiluminescence-based immunoassay performed on the Mindray CL-1200i Analyser (Mindray, China), following the manufacturer’s instructions. According to available manufacturer documentation and published evaluations, the assay has a reported sensitivity of approximately 95.6% and specificity of 99.2% [11]. Categorical variables were numerically coded and verified to ensure internal consistency before statistical analysis. Age was analysed both as a continuous variable and as a categorical variable (< 20, 20–30, 31–40, > 40 years). These categories were defined based on epidemiological relevance and to allow comparison with published studies on hepatitis C seroprevalence, reflecting distinct life stages associated with potential differences in exposure risk. Gender and year of diagnosis (2020–2024) were treated as categorical variables to assess demographic distribution and temporal patterns over the study period.

### Statistical analysis

The collected data were analysed using SPSS v29 (IBM SPSS Statistics, USA). Descriptive statistics were presented as frequencies and percentages for categorical variables. Continuous variables were expressed as means and standard deviations (SD). Group comparisons were conducted using independent-sample t-tests or one-way analysis of variance (ANOVA), as appropriate. Associations between categorical variables were assessed using the Chi-square test.

Exploratory univariate logistic regression analyses were initially performed to evaluate association between demographic characteristic and anti-HCV seroreactivity. Variables with *p* < 0.200 in the univariate analysis were subsequently included in an exploratory multivariate logistic regression model. Age and gender met this criterion and were retained in the final model. To account for possible temporal variation in anti-HCV seroreactivity during the study period, the adjusted analysis included year of diagnosis as a covariate. Given the limited number of anti-HCV-reactive individuals, the multivariable analysis was considered exploratory. The regression analysis was intended to identify patterns of association rather than establish robust independent predictors. Results were expressed as odds ratios (OR) or adjusted OR (AOR) with 95% confidence intervals (CI). Model fit was assessed using the Hosmer–Lemeshow goodness-of-fit test. All statistical tests were two-tailed, and a *p* < 0.05 was deemed statistically significant.

## Results

### Demographic characteristics related to anti-HCV reactivity

The demographic characterisation of the participants and anti-HCV reactivity are presented in Table 1. A total of 5,399 medical records were analysed. The age of the participants ranged from 1 to 91 years old, with a mean of 37.4±12.6 years. The most representative group were individuals aged 31–40 years old (39.1%, 2112/5399), females (64.6%, 3487/5399), and records from 2022 to 2024 (22.5% − 28.3%). Overall, 1.1% (57/5399) of individuals were anti-HCV reactive. The mean age of anti-HCV reactive individuals (47.5±15.7 years old) was higher than that of non-reactive individuals (37.3±12.5 years), with a difference of 10.2 years (*p* < 0.001). The proportion of anti-HCV reactivity among individuals up to 40 years ranged from 0.5 to 0.6%, whereas individuals over 40 years showed a proportion of 2.1%. A statistically significant association was observed between age group and anti-HCV reactivity (X^2^ = 23.8; df = 3; *p* < 0.001), with a small-to-moderate effect size (Cramer’s V = 0.1). Exploratory multivariate analysis showed lower odds of anti-HCV reactivity among individuals aged 20–30 years [AOR: 0.30 (95% CI: 0.14–0.65), *p* = 0.003] and 31–40 years [AOR: 0.31 (95% CI: 0.16–0.59), *p* < 0.001], compared with individuals over than 40 years. Anti-HCV reactivity was numerically higher among males (1.4%) than females (0.9%), but the difference was not statistically significant after adjustment [male: AOR: 1.26 (95% CI: 0.73–2.15), *p* = 0.407]. Regarding temporal distribution, the proportion of anti-HCV-reactive individuals remained relatively stable during the study period, ranging from 0.9% (8/869) in 2020 to 1.1% (17/1527) in 2024. Across the study period, the odds ratios did not show statistically significant differences [2021: AOR: 0.98 (95% CI: 0.32–3.02), *p* = 0.971; 2024: OR: 1.05 (95% CI: 0.45–2.46), *p* = 0.906].

**Table 1:** Demographic characteristics associated with hepatitis C virus infection in individuals from Luanda, Angola

Independent variables	*N* (%)	Hepatitis C Virus infection			Univariate analysis		Multivariate analysis	
		Non-reactive (%)	Reactive (%)	*P*-value	OR (95% CI)	*P*-value	AOR (95% CI)	*P*-value
Overall	5399 (100)	5342 (98.9)	57 (1.1)					
Age (years), Mean ± SD	37.4 ± 12.6	37.3 ± 12.5	47.5 ± 15.7	**< 0.001**				
Age distribution								
<20 yrs	207 (3.8)	206 (99.5)	1 (0.5)	**< 0.001**	0.23 (0.03–1.70)	0.150	0.24 (0.04–1.75)	0.158
20–30 yrs	1376 (25.5)	1368 (99.4)	8 (0.6)		0.28 (0.13–0.60)	**0.001**	0.30 (0.14–0.65)	**0.003**
31–40 yrs	2112 (39.1)	2099 (99.4)	13 (0.6)		0.30 (0.16–0.56)	**< 0.001**	0.31 (0.16–0.59)	**< 0.001**
>40 yrs	1704 (31.6)	1669 (97.9)	35 (2.1)		1.00		1.00	
Gender								
Female	3487 (64.6)	3457 (99.1)	30 (0.9)	0.058	1.00		1.00	
Male	1912 (35.4)	1885 (98.6)	27 (1.4)		1.65 (0.98–2.78)	0.060	1.26 (0.73–2.15)	0.407
Year of diagnosis								
2020	869 (16.1)	861 (99.1)	8 (0.9)	0.984	1.00		1.00	
2021	532 (9.9)	527 (99.1)	5 (0.9)		1.02 (0.33–3.14)	0.971	0.98 (0.32–3.02)	0.971
2022	1214 (22.5)	1200 (98.8)	14 (1.2)		1.26 (0.52–3.01)	0.609	1.19 (0.50–2.86)	0.695
2023	1257 (23.3)	1244 (99.0)	13 (1.0)		1.13 (0.46–2.73)	0.795	1.05 (0.43–2.55)	0.916
2024	1527 (28.3)	1510 (98.9)	17 (1.1)		1.21 (0.52–2.82)	0.656	1.05 (0.45–2.46)	0.906

Note: Bold numbers mean that the results were statistically significant for the chi-square test, independent-samples t-test and/or logistic regression (*p* < 0.05)

### Distribution of anti-HCV-reactive cases between 2020 and 2024

The distribution of anti-HCV-reactive cases over time is presented in Table 2. Among the 57 anti-HCV-reactive individuals, the proportion of cases among the total positives accounted for 14% (8/57) in 2020 and 29.8% (17/57) in 2024. The mean age of anti-HCV-reactive individuals varied from 48.1±19.1 years in 2020 to 46.4±17.7 years in 2024, with no statistically significant difference (*p* = 0.835). Descriptive variations in the distribution of cases across age groups were observed, including higher proportions among individuals aged under 20 years (0% to 5.9%), 20–30 years (12.5% to 23.5%) and over 40 years (50% to 64.7%), and a lower proportion among those aged 31–40 years (37.5% to 5.9%), however, these differences were not statistically significant (*p* = 0.495). Similarly, descriptive variations were observed in gender distribution, with the proportion of females decreasing from 75% to 47.1%, and that of males increasing from 25% to 52.9%, without statistical significance (*p* = 0.354).

**Table 2:** Proportion of HCV cases over time (2020–2024) in individuals from Luanda, Angola

Independent variables	*N* (%)	Years of HCV infection diagnosis (2020–2024)					*P*-value
		2020 (%)	2021 (%)	2022 (%)	2023 (%)	2024 (%)	
Overall	57 (100)	8 (14.0)	5 (8.8)	14 (24.6)	13 (22.8)	17 (29.8)	
Age (years), Mean ± SD	47.5 ± 15.7	48.1 ± 19.1	50.0 ± 21.4	50.8 ± 14.2	43.9 ± 10.9	46.4 ± 17.7	0.835
Age distribution							
<20 yrs	1 (1.8)	0 (0.0)	0 (0.0)	0 (0.0)	0 (0.0)	1 (5.9)	0.496
20–30 yrs	8 (14.0)	1 (12.5)	0 (0.0)	1 (7.1)	2 (15.4)	4 (23.5)	
31–40 yrs	13 (22.8)	3 (37.5)	3 (60.0)	3 (23.1)	3 (23.1)	1 (5.9)	
>40 yrs	35 (61.4)	4 (50.0)	2 (40.0)	10 (71.4)	8 (61.5)	11 (64.7)	
Gender							
Female	30 (52.6)	6 (75.0)	4 (80.0)	7 (50.0)	5 (38.5)	8 (47.1)	0.354
Male	27 (47.4)	2 (25.0)	1 (20.0)	7 (50.0)	8 (61.5)	9 (52.9)	

## Discussion

HCV infection remains a public health concern in sub-Saharan Africa, a region with high burdens of infectious diseases, limited healthcare infrastructure, and socioeconomic inequalities [2]. Our data collected between 2020 and 2024 in Luanda, the capital city of Angola, provides evidence regarding HCV seroprevalence and associated demographic characteristics in individuals attending a private clinical laboratory, in whom anti-HCV testing was requested by physicians due to clinical suspicion of liver disease. These findings contribute to a better understanding of the epidemiological profile of HCV in this setting.

The overall seroprevalence of anti-HCV observed in the present study was 1.1%, which is consistent with estimates reported in low-risk populations in other African countries [1]. A meta-analysis conducted by Rao et al. (2015) reported a pooled HCV seroprevalence of 2.98% in the general population of sub-Saharan Africa, highlighting substantial regional heterogeneity [12]. Between 2020 and 2024, a slight increase in the proportion of anti-HCV positive results were observed (0.9% to 1.1%). Although this variation was not statistically significance, it may reflect differences in testing patterns, healthcare access, or population composition over time rather a true temporal change in infection burden. This observation highlights the importance of continuous surveillance of HCV infection, particularly in settings where longitudinal population-based data remain limited. Similarly, previous studies have reported increasing HBV seroprevalence in Luanda between 2018 and 2022 [13]. Although HBV and HCV differ in transmission patterns, these findings highlight the importance of maintaining surveillance of viral hepatitis in Angola.

Age was associated with differences in anti-HCV seropositivity. Anti-HCV reactive individuals had a higher mean age compared to non-reactive individuals (47.5 ± 15.7 vs. 37.3 ± 12.5 years; *p* < 0.001). A higher proportion of seropositivity was also observed among individuals over 40 years of age (2.1%), compared with younger age groups (0.5–0.6%). Similar pattern have been reported in other African settings, including studies conducted among healthcare workers in Egypt, where higher seroprevalence was observed in older age groups [14]. This association between older age and HCV infection has been widely documented in African studies and is often linked to past medical exposures, such as transfusions or unsafe medical practices performed under low biosafety standards [15, 16]. Previous studies among migrants from sub-Saharan Africa have also reported higher HCV prevalence in older cohorts, which may reflect infections acquired earlier in life and remaining undiagnosed for extended periods [17, 18]. Descriptive variations in anti-HCV reactivity were observed among younger individuals (< 20 years). However, because behavioural and exposure-related data were not available, no conclusions can be drawn regarding possible transmission routes. Future studies should investigate behavioural and clinical factors associated with anti-HCV reactivity in younger age groups in the Angolan population.

It is worth mentioning that although the majority of participants were female (64.6%), a higher proportion of ant-HIV positivity was observed among males (1.4%) compared to females (0.9%). Although anti-HCV reactivity was numerically higher among males, the difference was not statistically significant after adjustment. Similar pattern has been reported in other settings [19], although the present study did not include information on behavioural or exposure-related factors that might explain these differences. However, stratified analysis showed that among the anti-HCV-positive women, the majority were older than 30 years (87%), with a smaller proportion (13%) aged 30 years or younger (results not shown). This pattern is consistent with the overall findings, showing higher HCV positivity in adult age groups regardless of gender. A study conducted in Ghana suggests that cultural practices such as tribal scarification, ritual circumcision, and home births performed by traditional birth attendants are strongly associated with increased HCV risk [20]. The cultural and geographic diversity of sub-Saharan Africa directly influences HCV transmission routes [3, 17].

HCV seropositivity over time showed variability across the study period, with reported proportion increasing from 14.0% in 2020 to 29.8% in 2024 among positive cases. These differences likely reflect changes in the composition of the tested population over time in urban population. Indeed, previous study carried out by our research team showed that HCV was related to the area of residence, with 48% of HCV positive cases observed in urban setting [9]. Previous studies among blood donors in Luanda reported anti-HCV seroprevalence estimates of 5.1% in 2016 [21] and 9.3% in 2023 [9]. It is also important to note that seroprevalence estimates from blood donor populations often underestimate the true prevalence in the general population due to selection bias, as donors are required to meet strict eligibility and exclusion criteria.

The mean age of HCV-positive individuals declined slightly from 48.1 to 46.4 years from 2020 to 2024, suggesting relative stability in age-associated risk. However, stratified analysis revealed an increase in the proportion of HCV seropositivity among individuals < 20 years (0.0% to 5.9%), 20–30 years (12.5% to 23.5%), and over 40 years (50.0% to 64.7%), and a decrease in the 31–40-year group (37.5% to 5.9%). The observed higher burden among younger cohorts could be related to factors such as unsafe cosmetic procedures, intravenous drug use, adverse sociodemographic conditions, and high-risk sexual behaviours, which warrant further investigation in future studies [22]. Regarding gender distribution, there was observed a decrease in the proportion of female seropositivity (75.0% to 47.1%) and an increase among males (25.0% to 52.9%), although no statistical significance (*p* = 0.354). These demographic changes may reflect alterations in sample composition and could help identify groups for more rigorous surveillance. However, population-based studies with confirmatory HCV RNA testing are needed before definitive screening priorities can be established, particularly regarding younger and male populations in this urban Angolan setting.

This study has some limitations. Firstly, the retrospective cross-sectional design limits the ability to establish causal relationships between demographic characteristics and anti-HCV-reactivity. Therefore, a longitudinal study would be necessary to confirm temporal patterns and incidence rates of anti-HCV reactivity. Secondly, HCV status was determined using anti-HCV antibody testing alone. This approach cannot distinguish active from resolved infection, and the absence of confirmatory HCV RNA or antigen testing may overestimate the prevalence of current viraemic infection. It also prevents assessment of infectivity, treatment eligibility, and clinical follow-up needs. Thirdly, important behavioural, clinical, and socioeconomic variables were not available, including history of medical procedures, traditional practices, blood transfusions, or injection exposures, limiting more detailed interpretation of transmission dynamics. Finally, individuals seeking care in private facilities may differ significantly from the general population in terms of socioeconomic status, access to health services, and healthcare-seeking behaviour, which can lead to overestimation or underestimation of HCV seroprevalence. Despite these limitations, the findings contribute to the limited available data on anti-HCV seroprevalence in Angola. These findings highlight the importance of improved surveillance systems and may help guide future anti-HCV screening strategies, which should be informed by population-based studies with confirmatory HCV RNA testing.

## Conclusion

This retrospective laboratory-based study found an overall anti-HCV reactivity of 1.1% among individuals tested at a private clinical laboratory in Luanda between 2020 and 2024. Anti-HCV reactivity was higher among individuals aged over 40 years, while gender differences were not statistically significant after adjustment. These findings should be interpreted with caution due to anti-HCV antibody testing without HCV RNA confirmation. The results support the importance of maintaining screening and surveillance activities in clinical settings, including integration with existing HIV/HBV programs, to strengthen HCV control and elimination efforts in Angola. Further population-based studies with HCV RNA confirmation and detailed behavioural, clinical, and socioeconomic data are needed to better characterise HCV epidemiology in Angola.

## Data Availability

Data is provided within the manuscript. The data supporting this study’s findings are available on request from the corresponding author.

## Abbreviations

ANOVA: Analysis of variance
CI: Confidence interval
CLIA: Chemiluminescence immunoassay
CNIC: National centre for scientific research
HCV: Hepatitis C virus
OR: Odds ratio
SD: Standard deviation
SSA: Sub–saharan Africa
WHO: World health organization
